# Transgenerational epigenetic inheritance: a critical perspective

**DOI:** 10.3389/freae.2024.1434253

**Published:** 2024-08-30

**Authors:** Adrian Bird

**Affiliations:** Wellcome Centre for Cell Biology, University of Edinburgh, Michael Swann Building, The King’s Buildings, Edinburgh, United Kingdom

**Keywords:** transgenerational epigenetic inheritance, epigenetics, acquired characteristics, epigenome analyses, epigenetics versus genetics

## Abstract

Transgenerational epigenetic inheritance in humans and other vertebrates has been controversial for over 150 years and remains so. It currently depends on the assumption that the environment is able to influence the genome, in particular by altering epigenetic marks such as DNA methylation, and that these acquired markings can enter the germline and affect phenotypes in the next generation. This article will offer a critical overview of some of the evidence beneath these assumptions–particularly regarding mammals. Whilst genome sequencing increasingly strengthens the causal relationship between genotypes and organismal characteristics, the evidence for many potentially important forms of environmentally induced epigenetic inheritance remains inconclusive.

## Introduction

Attempts to detect transgenerational inheritance of acquired characteristics date back centuries. Darwin argued against it and Weismann (who postulated the immortal germline in animals) devoted significant space in his books to debunking contemporary claims (Weismann, 1889). The pioneering *Drosophila* geneticist Thomas Hunt Morgan also had strong negative views on the subject:

“If we had time to pass in review of the many attempts that have been made during the last hundred years to re-establish Lamarck’s teaching, the story would reveal the weakness and futility of one attempt after another – a veritable nightmare of false logic, of insufficient evidence, of mistakes of many kinds and of sensationalism rampant.” (Morgan, 1932)

Today transgenerational epigenetic inheritance (TEI) retains a high profile and depends on two assumptions: 1) that the environment is able to instruct the epigenome, for example, by altering DNA methylation patterns; 2) that these acquired markings can be transferred to the germline and passed on to progeny with effects on the phenotype of the next generation. The definition of the word “epigenetics” has provoked much discussion and often specifically incorporates the requirement that non-genetic changes to the epigenome should be heritable. Here, however, epigenetics is considered to be “the structural adaptation of chromosomal regions so as to register, signal or perpetuate altered activity states” (Bird, 2007), covering the panoply of chemical and structural modifications to chromatin that comprise the epigenome. This commentary does not dwell on the difference between intergenerational and transgenerational inheritance, and it also makes reference to intragenerational effects. The implication of the distinctions between these categories for TEI has been reviewed elsewhere (Fitz-James and Cavalli, 2022). For a recent critical review of the field, see reference (Horsthemke, 2018).

In recent years the notion that a transient environmental effect can be memorized and go on to impact characteristics over multiple generations has become pervasive. It has been cast as a revolutionary way of seeing human inheritance that contradicted the conventional view that DNA sequence is its primary mediator (Carey, 2013). Traditional genetic explanations argue that many human characteristics are “hard-wired” in our genomes, with the dual implications that they are stochastic in origin and under normal circumstances irreversible. Refreshingly to some, the “epigenetics revolution” questions both of these features. Epigenetic mechanisms provide a potential conduit whereby the environment can alter the way our genes work. In principle, a specific external perturbation might lead to a response that is adaptive but whose inheritance would be “soft” – that is, potentially reversible. The contrast between genetic and epigenetic modes has updated the historical dichotomy of “Nature versus Nurture”, by paraphrasing key aspects as “Genetics versus Epigenetics”.

## Environmental effects on the epigenome?

While the notion that the environment can directly provoke epigenetic changes in somatic cells at least is quite widely accepted, the supporting evidence is often ambiguous. This is partly because of the difficulty in distinguishing whether epigenomic changes are causal or consequential. For example, exposure of cells to toxins can lead to changes in DNA methylation, but are these a direct response to the perturbation, or are they a consequence of programmed cellular responses to stress? The gene expression programme associated with a particular cell state evidently leaves a subtle but stable footprint in the pattern of DNA methylation. This underlies its utility as a diagnostic marker used in, for example, tumour categorisation, but the footprint may well arise downstream of the primary effect. There are well known mechanisms by which external stresses can trigger a physiological response, including induction of genes whose function is to cope with the disruption (e.g., heatshock, coldshock, infection, DNA damage, etc.). However, these represent evolved defence mechanisms, arising during evolution to respond to and nullify the effects of environmental perturbation. Programmed responses of this kind often affect the epigenome, but–as in other situations where gene expression switching occurs–transcription factors are the prime movers, with epigenetic marks behaving as their clients to consolidate, prolong or modulate corrective effects. Compatible with the notion that epigenetic changes are often secondary, we know that the presence of DNA binding proteins (e.g., those involved in transcription) can interfere with DNA methylation (Lin et al., 2000) and also that widely expressed TET enzymes collaborate with transcription factors to demethylate 5-methylcytosine (Zhang et al., 2023). Thus, while the epigenome is often affected, this does not appear to be the primary mediator of these physiological responses.

Twins provide the classic test of whether a trait is genetic or non-genetic (e.g., environmental) in origin. Genetically determined characters will always be more concordant between monozygotic twins (derived from a single fertilised egg) than dizygotic twins (derived from two independently fertilised eggs), whereas non-genetic effects are expected to be of similar frequency in both twin types. Despite the striking phenotypic similarity of monozygotic twins (colloquially known as “identical twins”), they have often been invoked to illustrate the importance of epigenetic influences on phenotype (Bell and Spector, 2011). The argument is that, since the two genomes are identical, any differences that do arise can be reliably attributed to environmental effects, perhaps on the epigenome. This logic turns out to be flawed, as genome sequencing shows that monozygotic twins acquire numerous genetic differences due to *de novo* mutations as they grow and develop (Jonsson et al., 2021). So, while sporadic differences between otherwise identical twins might be due to differing exposure to environmental influences, they could also be due to mutations arising after fertilization that affect one but not the other twin. A further possibility is that, even without environmental or genetic perturbation, developmental trajectories can vary stochastically. An extreme example is provided by mice deficient for the imprinting regulator TRIM28 (Dalgaard et al., 2016). Animals do not show continuous variability in body weight but are either obese or non-obese despite “identical” genotypes. In this case, phenotypic instability is greatly accentuated by a genetic mutation in the *Trim28* gene, but it illustrates the potential for a constant genome to give rise to distinct developmental outcomes.

## Darwinian transgenerational epigenetic inheritance

In invertebrates and plants there are clear examples where environmental stresses have transgenerational consequences (Fitz-James and Cavalli, 2022). For example, an RNA-based system of acquired immunity ensures that progeny of the invertebrate worm *Caenorhabditis elegans* are born with pre-existing resistance to local pathogens (Ashe et al., 2012). The evolution of this mechanism confers an obvious selective advantage which can be viewed as a conventional Darwinian response to an evolutionary pressure. It has been noted previously (Bird, 2013) that human mothers strive to achieve an analogous benefit via colostrum which transmits antibodies directly to the new-born progeny. As in the worm, this mechanism is presumably a response to the selective pressure to confer acquired parental immunity upon offspring, although in mammals it is not usually considered to be an example of TEI. An intriguing addition to this type of stress response is seen in the yeast *Schizosaccharomyces pombe*, which survives treatment by a variety of toxins through heterochromatic silencing of key genes (Torres-Garcia et al., 2020). Though dependent on chromatin-mediated repression via changes to the epigenome, this process is unstable rather than heritable, as it dissipates rapidly once the toxin is removed. There are potential scenarios whereby transient epigenetic effects of this kind might be selectively advantageous, for example, by allowing time for genetic or other physiological adaptations to arise. Robust evidence for equivalent mechanisms in mammals is absent. It is in any case questionable whether such bet-hedging strategies could be beneficial in relatively small populations.

Apart from a few evolved adaptive mechanisms, most transgenerational consequences of stress appear to be random rather than an attempt to correct the effects of an insult. In this respect they resemble genetic mutations, which are blind to selective advantage or disadvantage. In plants, for example, many transmitted epigenetic changes seem directionless rather than adaptive (Heard and Martienssen, 2014). The intrinsic heritability of epigenetic modifications is also in doubt. Whilst impressive transgenerational transmission of histone marks in *C. elegans* and *S. pombe* have been reported, this was only apparent in mutants that lack the ability of the wildtype organisms to remove these marks (Audergon et al., 2015; Katz et al., 2009). As mentioned above, adaptive resistance to toxins in wildtype *S. pombe* is rapidly lost when the agent is removed (Torres-Garcia et al., 2020). A related phenomenon has been seen in mice, where transgenerational inheritance of epigenetic marks is only observed when the *Dppa2* gene is mutated (Carlini et al., 2022). The authors propose that DPPA2 protein acts as a safeguard against intergenerational transmission of epigenetic information. It seems that, rather than eagerly harnessing environmental inputs from the life experiences of their ancestors via epigenetic mechanisms, organisms strive to prevent contamination of a new generation with the accumulated epigenetic baggage of the previous one.

## The quest for reliable TEI in mammals

Despite the best efforts of mammalian parents and offspring to remove epigenetic marks, there are cases where they are reproducibly subject to transgenerational leakage. The best characterised example is the Agouti mouse, where variable DNA methylation at a transposon inserted near a coat colour gene shows modest but convincing heritability (Morgan et al., 1999). In principle this system allows testing of the hypothesis that the environment can induce or modulate TEI. An early report claimed that diet produces heritable changes in coat colour of Agouti mice (Wolff et al., 1998), suggesting a direct effect of the environment on gene activity via the epigenome. However, subsequent studies with a larger sample size at this and other loci failed to validate this finding (Rosenfeld, 2012; Bertozzi et al., 2021). As transposons or their inactive remnants are highly abundant in mammalian genomes, the Agouti phenomenon raised the possibility that variable transposon methylation could lead to epigenetic inheritance in a similar way. However a screen for other equivalent loci in mice was largely negative (Kazachenka et al., 2018). The Agouti locus cannot therefore be viewed as a paradigm for TEI in mammals; its ability to transmit epigenetic information between generations–albeit inefficiently–is rare.

There is as yet no validated mechanism by which the external environment can directly communicate with the epigenome. The notion that some form of informational RNA can carry the memory of environmental constraints via mammalian sperm has proponents (Conine and Rando, 2022), but so far lacks robust experimental validation. In mammals, DNA methylation itself is largely stripped from the gametic and early embryonic genomes (Luo et al., 2018), a fact that weakens its credentials as a potential carrier of intergenerational information. An intriguing recent exception to this conclusion involves the stable transmission of DNA methylation at two CpG islands through multiple generations in mice (Takahashi et al., 2023). Interestingly, this heritable DNA modification was triggered by a genetic deletion and could not be reproduced by simply adding DNA methylation without the initiating mutation (Horsthemke and Bird, 2023). Whether a purely environmental perturbation could generate a heritable epigenetic response of this magnitude is unclear.

Regardless of mechanistic uncertainty, the quest for environmentally induced TEI has continued unabated. Numerous reports have traced the long-term consequences of various insults, including chemical exposure, maternal deprivation, restraint and dietary deprivation, in mice or rats. For example, several studies reported effects on gene expression in the progeny of severely protein-deprived male mice, but these were indetectable in the second generation (Radford et al., 2014; Carone et al., 2010). The results suggest that environmental effects on the next generation in mammals are short-lived. In contrast, the effects of some treatments are reported be transmissible over many generations. One prominent perturbation involves administration of endocrine disruptors to rats, where multi-generational effects have been reported (Anway et al., 2005), but not always replicated (Schneider et al., 2013). Notably, many of these studies involve outbred–and therefore genetically somewhat heterogeneous–rats, making it difficult to conclusively rule out the possibility that selection of genetically more toxin-resistant germ cells underlies heritability. However, a recent report provides robust evidence for transmission of the consequences of an environmental insult between generations. The study shows that sperm from male mice in which the microbiome has been pharmacologically ablated trigger reduced placental size and some progeny mortality when used to fertilise untreated females (Argaw-Denboba et al., 2024). In their search for an underlying mechanism, the authors showed that this effect is highly unlikely to be transmitted via the sperm epigenome. A speculative alternative explanation is that the effect on placental growth is a response by the mother to perceived defects in, or negative signals carried by, sperm from microbiome-free males which lead her to withhold placental resources. It remains to be seen whether this study has uncovered a novel mechanism of reproductive quality control or if some other process is at work.

## Questionable evidence for TEI in humans

The controversy that often surrounds evidence for TEI in mammals also extends to related phenomena in humans (Fitz-James and Cavalli, 2022; Daxinger and Whitelaw, 2010). One study that has achieved almost iconic status in the field concerns the Swedish famine (Pembrey et al., 2006) which purports to show inheritance of disease susceptibility by grandchildren of those who underwent the trauma of starvation. Unfortunately, this study–and others like it–ignored the potential effects of cultural transmission of behaviours within families. This omission is highlighted by a report that progeny of individuals who experienced starvation during the siege of Leningrad in 1941 tended to display significantly different eating patterns compared with controls, including “excessive red meat consumption” (Tolkunova et al., 2023). It is surely conceivable that cultural influence in the home played a role here. Also of concern is the report that replication of the Swedish famine study using a much larger cohort failed to reproduce paternal grandfather effects on diabetes/heart disease which were prominent in the original study (Vagero et al., 2018). A more general criticism of such analyses is that they often suffer from “multiple testing” (Kevin Mitchell, http://www.wiringthebrain.com/2018/05/grandmas-trauma-critical-appraisal-of.html?spref=tw), which refers to the risk that, if enough correlations are sought in a dataset, some will eventually be found just by chance.

Whereas organisms usually seek to repair and correct damage, it is striking that most examples of putative TEI in humans involve transmission of serious defects, such as acquired obesity, diabetes, or impaired learning and memory [all reported to result from ancestral environmental stress (Rando, 2012)]. Why should organisms seek to perpetuate disease susceptibility within their lineage unless the defect is outweighed by some emergent advantage? For comparison, heterozygosity for genetic mutations that cause sickle cell anaemia confers beneficial resistance to malaria (Allison, 1954). It has been argued that epigenetic changes could likewise create a favourable cost-benefit outcome. As an example, parents may seek to epigenetically adapt the metabolism of their offspring to an anticipated environmental stress, such as food shortage if they themselves are experiencing starvation (Hales and Barker, 2001). Against this, it can be argued that in long-lived mammals like humans this could be a risky prediction that would backfire if the famine is short-lived. Indeed, the Barker hypothesis argues that a surfeit of food intake in adulthood increases the likelihood of metabolic disease for an individual who experienced starvation *in utero*.

Despite reservations about the supporting evidence, the notion that TEI in humans is both real and important has gained widespread traction as an acceptable explanation for human variability. Accordingly, claims that trauma has long-term effects on the health of descendants often find their way into the popular press. Exemplifying this, a report that exposure to the horrors of the Holocaust during the second world war had adverse consequences for the children of survivors (Yehuda et al., 2016) made headline news, despite numerous technical weakness (e.g., a sample of only 32 people, limited controls and unclear methodology/interpretation). The TEI field is also sustained by a seemingly insatiable appetite in the scientific literature. From a sociological perspective it is remarkable that a concept that has struggled to gain acceptance for over a hundred years can constantly refresh its appeal as a disruptive alternative to conventional wisdom.

## Genetics or epigenetics or something else?

The idea that human (and other mammalian) phenotypes can be heritably altered by direct effects of the environment on gene activity has been controversial for centuries and remains so. If we exclude known “Darwinian” phenomena that happen to involve epigenetics, such as pathogen resistance in *C. elegans*, it seems that the influence of the environment via the epigenome is limited and transmission of epigenetic information across generations is often weak and temporary. Why then has TEI been able to stubbornly retain the aura of a revolutionary anti-establishment wave about to break? A potential driver is cultural resistance to the deterministic view–illustrated by the extreme similarity of “identical twins” – that human characteristics are predominantly hard-wired in the genome. Fortunately, while epigenetic phenomenology has been accumulating, genetic understanding has been progressing fast and many medical conditions whose origin was previously unknown can now be attributed unambiguously to mutations in specific genes. For example, the aetiology of intellectual disability (often accompanied by developmental delay) was for many years unknown, but genome sequencing has uncovered hundreds of causal mutations (Deciphering Developmental Disorders Study et al., 2017). In a notable recent case, longitudinal studies on an unprecedented scale provided a compelling causal link between the relatively common disorder multiple sclerosis and infection by Epstein-Barr virus (Bjornevik et al., 2022). Evidence suggests that the host immune response to proteins encoded by the viral genome coincidentally creates antibodies that can attack normal nerve cells (Lanz et al., 2022). Studies like these mean that the number of mystery diseases whose origin could hypothetically be attributed to epigenetic errors is falling as robust genetic explanations are uncovered. Interestingly, DNA sequence-based diagnosis, which has in the past been predominantly paediatric, is increasingly being extended to include adults with longstanding medical conditions. This has already shown that a high proportion of individuals whose disorders had been attributed to trauma at birth or other life experiences turned out to harbour well-established causal mutations (Langenfeld et al., 2021). The implication is that these conditions had been misdiagnosed as environmental when in fact they are genetic in origin. Despite spectacular progress in human genetics, it remains true that many disorders cannot be categorised as genetic despite DNA sequence data. One potential reason is that these phenotypes depend on a combination of multiple weaker mutant alleles. Hinting at this is the growing success of “polygenic risk scores”, which use the presence of natural variants at multiple risk loci as predictors of future morbidity (Torkamani et al., 2018). Regardless of the encroachment of human genetics, it is of course possible that many diseases will remain unexplained and could therefore, in theory at least, have a non-genetic cause.

## Concluding remarks

A major question remains: are there mechanisms in mammals that allow adaptations in response to external influences to be transmitted from one generation to the next? While there is some evidence that severe environmental insults can impact the succeeding generation, there is little reason to believe that these consequences are adaptive. Susceptibility to disease, which is presumably almost always maladaptive, would nevertheless be of interest, but here the magnitude and persistence of the effects reported so far is inconclusive. Perhaps the field of acquired immunity will be an interesting place to look. Might the potentially gigantic human datasets resulting from the recent SARS Covid 2 pandemic identify non-genetic ways of passing on resistance or susceptibility? Of course, the answer may be no.

## Data Availability

The original contributions presented in the study are included in the article, further inquiries can be directed to the corresponding author.
